# Convulsions

**Published:** 1891-02

**Authors:** 


### CONVULSIONS.

Not frequently convulsions occur in infancy in consequence of some internal difficulty of a temporary nature, and are never repeated in after life. But where they are of frequent occurrence in childhood there are grounds to fear that the sufferer will sooner or later become epileptic. Indeed, a large proportion of these troubles may be traced to the frequency of infantile convulsions. It is very difficult to discriminate between those early attacks, which are simply accidental, and not likely to recur, and those which are but the beginning of a life-long epilepsy. Hence, it is always requisite that the utmost care should be taken to prevent their recurrence. It is doubtless true, that in many instances children born with an epileptic tendency are cured of it by the intelligent care and nursing of parents, whereby their bodily weaknesses are strengthened, and their entire nervous system greatly changed for the better, even to a state of successful resistance of the threatened evil. All parents are under a serious responsibility in respect to all matters affecting the present good health and future well being of their natural offspring.

